# A Comparison of the Sensititre MycoTB Plate, the Bactec MGIT 960, and a Microarray-Based Molecular Assay for the Detection of Drug Resistance in Clinical *Mycobacterium tuberculosis* Isolates in Moscow, Russia

**DOI:** 10.1371/journal.pone.0167093

**Published:** 2016-11-30

**Authors:** Elena Y. Nosova, Danila V. Zimenkov, Anastasia A. Khakhalina, Alexandra I. Isakova, Ludmila Y. Krylova, Marina V. Makarova, Ksenia Y. Galkina, Maria A. Krasnova, Svetlana G. Safonova, Vitaly I. Litvinov, Dmitry A. Gryadunov, Elena M. Bogorodskaya

**Affiliations:** 1 The Moscow Research and Clinical Center for Tuberculosis Control of the Moscow Government Health Department, Moscow, Russia; 2 Engelhardt Institute of Molecular Biology, Russian Academy of Sciences, Moscow, Russia; St Petersburg Pasteur Institute, RUSSIAN FEDERATION

## Abstract

**Background:**

The goal of this study was to compare the consistency of three assays for the determination of the drug resistance of *Mycobacterium tuberculosis* (MTB) strains with various resistance profiles isolated from the Moscow region.

**Methods:**

A total of 144 MTB clinical isolates with a strong bias toward drug resistance were examined using Bactec MGIT 960, Sensititre MycoTB, and a microarray-based molecular assay TB-TEST to detect substitutions in the *rpoB*, *katG*, *inhA*, *ahpC*, *gyrA*, *gyrB*, *rrs*, *eis*, and *embB* genes that are associated with resistance to rifampin, isoniazid, fluoroquinolones, second-line injectable drugs and ethambutol.

**Results:**

The average correlation for the identification of resistant and susceptible isolates using the three methods was approximately 94%. An association of mutations detected with variable resistance levels was shown. We propose a change in the breakpoint minimal inhibitory concentration for kanamycin to less than 5 μg/ml in the Sensititre MycoTB system. A pairwise comparison of the minimal inhibitory concentrations (MICs) of two different drugs revealed an increased correlation in the first-line drug group and a partial correlation in the second-line drug group, reflecting the history of the preferential simultaneous use of drugs from these groups. An increased correlation with the MICs was also observed for drugs sharing common resistance mechanisms.

**Conclusions:**

The quantitative measures of phenotypic drug resistance produced by the Sensititre MycoTB and the timely detection of mutations using the TB-TEST assay provide guidance for clinicians for the choice of the appropriate drug regimen.

## Introduction

Drug-resistant tuberculosis [1] is difficult to cure and requires time-consuming (18–24 months), complex and expensive therapy accompanied by serious side effects [2–5]. The treatment outcome significantly depends on the rapid determination of the *Mycobacterium tuberculosis* (MTB) resistance profile to the maximal number of anti-tuberculosis drugs. Susceptibility can vary within wide limits, and in some cases, the dosage of a drug can be adjusted to overcome “borderline” or “moderate” resistance [6, 7]. In addition to the “gold standard” agar proportion method, various approaches utilizing the Bactec MGIT 960 (MGIT), Sensititre MycoTB (MycoTB), and Alamar Blue platforms have been developed for the quantitative characterization of resistance [8–15]. MycoTB is an inexpensive method to simultaneously identify the minimal inhibitory concentrations (MICs) of twelve first- and second-line drugs. Previous studies have demonstrated the method’s good reliability and reproducibility for most drugs compared with the agar proportion method [8, 11, 16] and the MGIT method [8, 17]. In this study, we compared these two phenotypic methods using clinical strains isolated in the Moscow region of Russia.

Previous research on the mechanism of MTB drug resistance development demonstrated correlations between certain mutations in the mycobacterial genome and the resistance levels [10, 13, 18]. Recently, the TBNET and RESIST-TB networks claimed that the identification of mutations in the *rpoB*, *katG*, *inhA*, *embB*, *rrs*, *rpsL* and *gyrA* genes of MTB clinical isolates had implications for the management of TB patients and complemented drug susceptibility testing (DST) [19]. Molecular genetic methods are attractive because they are rapid and can predict the resistance level from genetic data, thereby allowing personalization of prescription drugs within a few days of the diagnosis.

In the present study, we used a microarray-based TB-TEST molecular assay to characterize the mutations detected in clinical MTB strains and their correlations with resistance to rifampin (RMP), isoniazide (INH), ethambutol (EMB), fluoroquinolones (FQs), and second-line injection drugs (SLID) [20]. This assay allows the detection of an extended range of genetic determinants of resistance and establishes the genotypes of the most endemic strains in the Russian Federation. Recently, the TB-TEST assay has been approved by the Federal Service for Surveillance in Healthcare of the Russian Federation (Roszdravnadzor, www.roszdravnadzor.ru) for application in the laboratory diagnostics of MTB.

The objectives of this study were to compare the consistency of the three assays in the determination of drug resistance of clinical MTB isolates and to identify their resistance profiles using phenotypic and genotypic methods. The data obtained determined the conditions of the application of each method in a centralized mycobacterial laboratory.

## Materials and Methods

### Experimental settings and clinical strains

One hundred forty-four clinical MTB strains isolated in Middlebrook 7H9 liquid medium using an automated Bactec MGIT 960 system (Becton Dickinson, Sparks, MD, USA) were selected for this study. The strains were obtained from diagnostic material from TB patients at the Moscow Research and Clinical Center for Tuberculosis Control. Sequential isolates with resistance to either RMP or INH obtained during 2014 were analyzed. Additionally, 23 randomly selected isolates susceptible to both RMP and INH, which are typically tested only during susceptibility to first-line drugs following the Center protocol, were included in the study as a reference pool for the statistical calculations. In this selection, 67 samples were obtained from patients with primary pulmonary tuberculosis and 77 samples were isolated from previously treated patients (relapse and re-treatment cases). The study was approved by the Ethics Committee of the Moscow Government Health Department. The Ethics Committee waived the need for patient consent because the study did not include any personal identifiers and the samples were analyzed anonymously.

### Drug susceptibility testing

The MTB DST for streptomycin (STR), pyrazinamide (PZA), RMP, INH, EMB, ofloxacin (OFX), moxifloxacin (MFX), kanamycin (KAN), amikacin (AMK), PAS, and ethionamide (ETH) was performed using a Bactec MGIT 960 as previously described [21, 22].

The determination of the MIC using the Sensititre MycoTB Plate was performed as previously described [11, 16]. MTB growth in the wells with drug was evaluated visually using the mirror in comparison with the MTB growth in a drug-free control well on days 14–21. The MIC of a drug was considered the lowest concentration able to inhibit the visible growth of a microorganism in a well. The resistance breakpoints were based on previously established concentrations [STR– 2.0, RMP– 1.0, rifabutin (RFB)– 0.5, INH– 0.2, EMB– 5.0, OFX– 2.0, MFX– 0.5, KAN–>5.0, AMK–>4.0, PAS– 2, ETH– 5, and D-cycloserine– 30 μg/ml] [17]. A correlation analysis of the MICs for pairs of drugs was performed using the Statistica software version 10 (Dell Software, Tulsa, OK, USA).

### Mutation detection using the microarray-based TB-TEST molecular assay

DNA extraction from the MTB cultures obtained in the BACTEC MGIT 960 system was performed using a robot-aided Freedom EVO station (TECAN, Switzerland) and an M-Sorb DNA isolation kit (Syntol, LLC, Russia). The PCR and hybridization on the microarrays were performed as previously described [20]. The TB-TEST assay allows the detection of 28 substitutions in *rpoB*, 11 in *katG*, 5 in *inhA*, 5 in *ahpC*, 15 in *gyrA*, 23 in *gyrB*, 4 in *rrs*, 6 in *eis* and 23 in *embB* and established the lineages of the strains most endemic to the Russian Federation (Beijing, Haarlem, LAM and Ural).

## Results

### Comparison of the Sensititre MYCOTB with the Bactec MGIT 960

According to the DST results of the 144 MTB clinical isolates obtained using MGIT, 40 were extensively drug resistant (XDR; 28%), 65 were multidrug resistant (MDR; 45%), 18 were poly- and mono-resistant (DR) isolates (13%) and 21 were pan-susceptible (15%). Analysis of resistance to the first-line anti-tuberculosis drugs STR, PZA and EMB showed heterogeneity of these groups (Table 1). The most distinct representatives of the DR group were 3 isolates resistant to four of the five first-line drugs (susceptible to RMP) and 2 isolates mono-resistant to INH or the FQs. Two strains from the pan-susceptible group were resistant to STR. In total, 32 different resistance profiles were identified by analyzing the resistance to seven drugs.

**Table 1:** Characterization of isolate drug resistance using the Bactec MGIT 960 platform.

	Resistance profile against RMP-INH-FQs-SLID	Number of isolates	Additional resistance profile against STR-PZA-EMB and number of corresponding isolates					
			R-R-R	R-R-S	R-S-R	R-S-S	S-R-S	S-S-S
XDR, N = (40)	R-R-R-R	40	32	5	2	1		
MDR, N = (65)	R-R-R-S	9	5	2	2			
	R-R-S-R	28	12	5	8	2		1
	R-R-S-S	28	14	5	1	6		2
Drug-resistant, N = (18)	R-S-R-R	1						1
	S-R-R-R	2		1			1	
	S-R-R-S	1	1					
	S-R-S-R	2	1			1		
	S-S-R-R	1						1
	R-S-S-S	1				1		
	S-R-S-S	9	1	1		6		1
	S-S-R-S	1						1
S, N = (21)	S-S-S-S	21				2		19

The comparison of the phenotypic DST results using MGIT and MycoTB is summarized in Table 2. Of the 12 drugs available on the MycoTB plate, no comparisons were performed for RFB and D-cycloserine. Both methods had correlations above 90% for most of the drugs. The worst correlations between MGIT and MycoTB were observed for KAN and ETH; if the lower value of the breakpoint concentration for KAN on MycoTB was used and 20 strains with MICs of 5 μg/ml (which were resistant according to the MGIT method) were also considered resistant on the MycoTB, a correlation coefficient of 0.97 and Cohen’s kappa of 0.93 were achieved (compared to 0.83 and 0.65, respectively).

**Table 2:** MICs based on the comparison of the MycoTB plate and MGIT 960 results.

MGIT 960			MYCOTB											7H10/ 7H11 CC, μg/ml	Raw agreement	Cohen’s Kappa
Critical concentration (CC)	Result	No. of strains	Determined MIC (μg/ml) and no. of isolates ^a^									No. of resistant	No. of susceptible			
***Streptomycin***			0.25	0.5	1	2	4	8	16	32	>32					
1 μg/ml	R	117			2	3	13	14	5	7	73	112	5	2	0.97	0.89
	S	27	16	6	4	1						0	27			
***Rifampin***			0.13	0.25	0.5	1	2	4	8	16	>16					
1 μg/ml	R	107					2	1	3	2	99	107	0	1	1.00	1.00
	S	37	27	4	4	2						0	37			
***Isoniazid***			0.03	0.06	0.13	0.25	0.5	1	2	4	>4					
0.1 μg/ml	R	119			2	1	2	4	17	5	88	117	2	0.2	0.98	0.93
	S	25	14	10		1						1	24			
***Ethambutol***			0.5	1	2	4	8	16	32	>32						
5 μg/ml	R	79			1	11	52	13	2			67	12	5	0.90	0.79
	S	65	9	15	16	22	3					3	62			
***Ofloxacin***			0.25	0.5	1	2	4	8	16	32						
2 μg/ml	R	54				2	17	16	13	6		52	2	2	0.99	0.97
	S	90	25	39	23	3						0	90			
***Moxifloxacin***			0.06	0.13	0.25	0.5	1	2	4	8						
0.25 μg/ml	R	55			2	5	12	11	14	11		48	7	0.5	0.95	0.89
	S	89	29	18	37	5						0	89			
***Kanamycin***			0.63	1.25	2.5	5	10	20	40	>40						
2.5 μg/ml	R	73			5	20	19	6	23			48	25	5	0.83	0.65
	S	71	24	34	13							0	71			
***Amikacin***			0.13	0.25	0.5	1	2	4	8	16	>16					
1 μg/ml	R	35			1	2	4		6	22		28	7	4	0.95	0.86
	S	109	16	44	33	15	1					0	109			
***PAS***			0.5	1	2	4	8	16	32	64	>64					
4 μg/ml	R	31			3	3	8	9	3	3	2	28	3	2	0.94	0.83
	S	98	51	32	10	4		1				5	93			
***Ethionamide***			0.31	0.63	1.25	2.5	5	10	20	40	>40					
5 μg/ml	R	71				7	16	26	6	9	7	48	23	5	0.79	0.59
	S	58	1	5	14	10	24	3	1			4	54			

^a^ Vertical bold line splits susceptible and resistant isolates based on the critical concentrations established for the agar proportion method.

A pairwise comparison of the MICs revealed strong correlations between the OFX and MFX (r_xy_ = 0.86) and the KAN and AMK (r_xy_ = 0.88) resistance levels; these pairs of drugs share resistance mechanisms (S1 and S2 Tables). Elevated correlation levels were also observed for the first-line drugs (r_xy_ = 0.46), whereas the average correlation for all drugs was r_xy_ = 0.23. Additionally, elevated levels were observed for pairs of the second-line drugs (FQs and SLID and FQs and PAS). This observation likely reflects the joint use of FQs with SLID and FQs with PAS and the rare use of PAS with KAN for treatment traced in the history of the “successful” TB clones selected for this study. The average correlation between the second- and first-line drugs was 0.19 with the exception of the marked MIC correlations for RFB and RMP (r_xy_ = 0.49) and RFB-STR (r_xy_ = 0.36).

### Identification of mutations associated with drug resistance

**Fig 1:**
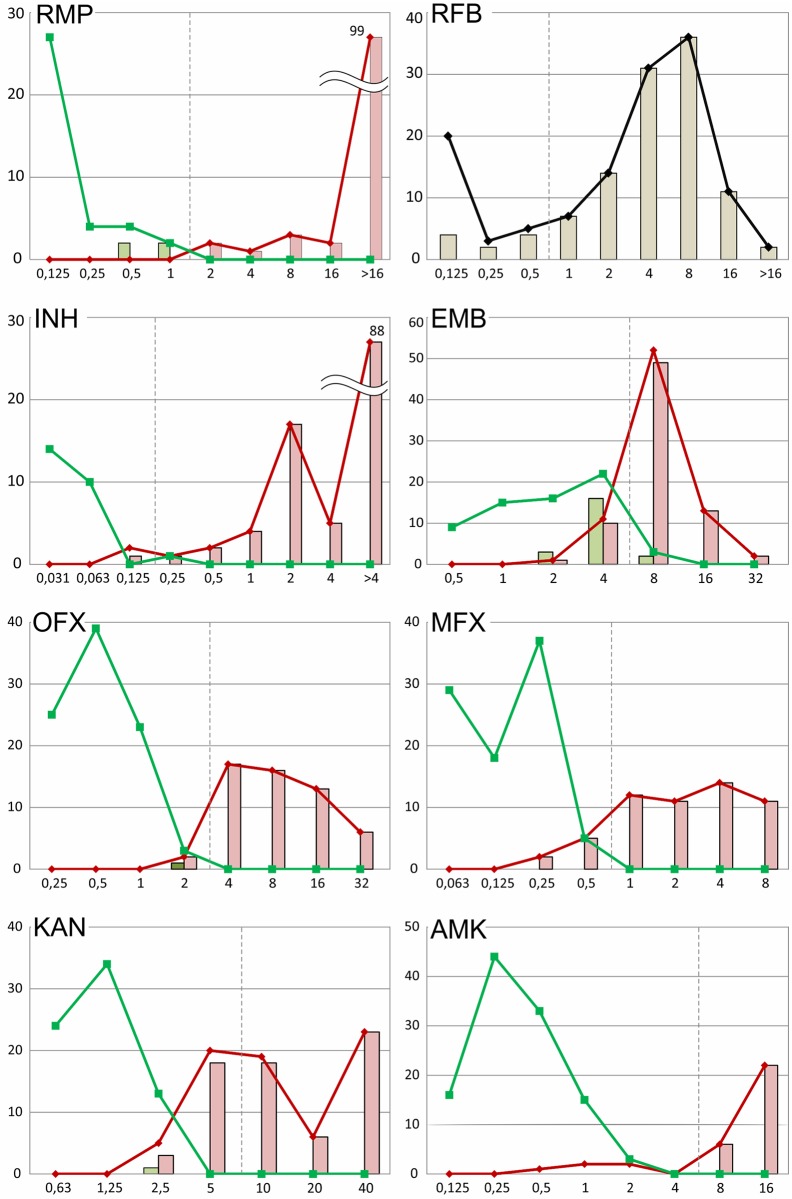
MIC distributions of the clinical isolates characterized using the MGIT and TB-TEST assays. Resistant and susceptible isolates based on the MGIT results are indicated by the red and green lines, respectively. The light-red and light-green bars represent the numbers of resistant and susceptible isolates with mutations detected by the TB-TEST. The MGIT was not performed for rifabutin (RFB); therefore, only the distributions of all isolates and the isolates with mutations are shown.

Genetic determinants of drug resistance were detected in the investigated MTB strains using a microarray-based TB-TEST molecular assay. The obtained data and performance characteristics including the correlation between the three assays are summarized in Fig 1 and S3 Table.

The analyzed isolates had 10 types of single mutations leading to substitutions in codons 516, 526 and 531 of the *rpoB* gene, which resulted in resistance to RMP; only one strain had a double substitution (L511P D516G). In total, mutations were identified in 111 strains, of which 4 strains were susceptible by both phenotypic methods. The S531L substitution was detected in most cases and was found in 93/111 (83.8%) strains. All MTB strains containing that mutation were resistant by MGIT, and most had a high resistance level by MycoTB. Only one strain had a borderline MIC of 2 μg/ml. Six strains with mutations in codons 516 and 526 had intermediate MICs of 0.5 and 1.0 μg/ml and low resistance MICs of 2.0 and 4.0 μg/ml, whereas 9 more isolates possessed the maximally detected MIC of >16 μg/ml.

For RFB, the MIC distribution had a maximum of 4–8 μg/ml; however, two of the MTB strains with the S531L substitution had MICs >16 μg/ml. In total, 10 MTB strains with mutations in *rpoB* were detected as susceptible to RFB by MycoTB, of which 4 strains had the S531L substitution.

Three MTB strains with substitutions in the 526 codon (H to L or N) had intermediate or low resistance to RMP (0.5–4 μg/ml) and were susceptible to RFB (0.125–0.25 μg/ml). Seven MTB strains containing substitutions in the 526 codon to R, Y, D and P had high MICs to both RMP (>16 μg/ml) and RFB (4–16 μg/ml).

Resistance discrepancies were observed for strains with the 516 codon substitutions for both RMP and RFB (N = 5). Strains with the double substitution (L511P D516G) were resistant to both RMP and RFB, with an intermediate RFB MIC of 2 μg/ml.

Among the mutations connected with INH resistance, the S315T1 substitution in *katG* was found in most cases and was detected in 110 of the 118 (93%) INH-resistant MTB strains identified by MGIT. Most of these isolates (N = 84) had high resistance levels (MIC >4 μg/ml). Thirty-five of the 110 strains had mutations in *katG* or in *katG* and *inhA* in addition to S315T1. One strain had a double mutation in *katG* and a c(-15)t mutation in *inhA*.

The *rpoB* S531L and *katG* S315T substitution combination was detected in 75% of the XDR and 88% of the MDR isolates. Double and triple mutations leading to INH resistance were identified in 14% (2/14) of the INH-resistant MTB DR strains, 23% (15/65) of the MDR strains and 45% (18/40) of the XDR strains.

The distributions of the MICs of the EMB-susceptible and resistant isolates detected by MGIT had two broad overlapping peaks with maxima at 4 and 8 μg/ml (Fig 1). Sixty-one percent of the strains (88/144) had MICs of 4 or 8 μg/ml. Fifteen strains had high levels of resistance (MICs of 16 or 32 μg/ml). Moreover, these strains were all determined to be resistant by MGIT and had mutations in the 306, 319, 354, 406 and 497 *embB* codons.

Strains with mutations in *embB* had different MICs for EMB (from 2 to 32 μg/ml), and 22% of these strains were susceptible by MGIT. The M306V substitution was the most prevalent mutation and was identified in 29 strains. Nine of these strains were detected as susceptible by MycoTB (MICs of 2–4 μg/ml); six of the 9 strains were detected as resistant, and 3 of the 9 were detected as susceptible by MGIT. The D354A substitution was the second most frequent mutation and was found in 15 strains, of which 3 strains were detected as susceptible by both methods. One additional isolate was detected as resistant by MGIT but had an MIC below the threshold.

A distinctive difference between resistance and susceptibility was observed for the OFX isolates using MycoTB. Most of the resistant isolates identified by MGIT (N = 52 of 54) had MICs ranging from 4 to 32 μg/ml, and 87 of the 90 MGIT-susceptible isolates had MICs in the range of 0.25–1 μg/ml. Five isolates with borderline MICs of 2 μg/ml (susceptible by MycoTB) were identified as either resistant (N = 2) or susceptible (N = 3) by MGIT. The D94A and double S91P D94A substitutions were identified in two resistant isolates. The *gyrB* N538K substitution was identified in one susceptible isolate, and no mutations were identified in 2 of the isolates. The N538K substitution was also identified in an isolate resistant by both MGIT and MycoTB that had an MIC of 4 μg/ml. The *gyrA* D94A substitution was mainly found in the resistant isolates (N = 6), with MICs ranging from 2 to 8 μg/ml.

Eight isolates resistant to OFX contained substitutions in the B subunit of DNA gyrase. *gyrB* mutations not accompanied by *gyrA* mutations were detected in 3 isolates. The D500H substitution with an MIC of 4 μg/ml was identified in one sample in addition to the two isolates mentioned earlier. Five other isolates also bore various substitutions in *gyrA*, with MICs ranging between 4 and 16 μg/ml.

The MIC distribution of the MFX-resistant (MGIT) strains ranged from 0.25 μg/ml to 8 μg/ml; thus, 7 isolates were susceptible according to MycoTB with MICs of 0.25 and 0.5 μg/ml (Fig 1). The following substitutions were detected in these isolates: D94A (N = 3); D94G; D94V; S91P D94A; and *gyrB* D500H. Strains with the D94G substitution predominately had MICs of 4 and 8 μg/ml, and two more isolates with the D94A substitution and one with D94V were resistant with MICs of 1 and 2 μg/ml. Other strains with MICs of 0.25 (N = 37) and 0.5 μg/ml (N = 5) were determined to be susceptible by MGIT, and no mutations were identified.

Mutations in the *rrs* 1400 region or in the *eis* promoter were found in 93% of the KAN-resistant isolates identified by MGIT; a total of 98% of the KAN-resistant isolates by MycoTB had MICs ranging from 10 to 40 μg/ml (breakpoint– 5 μg/ml). Twenty strains with an MIC equal to 5 μg/ml, which corresponded to susceptibility, were resistant by MGIT, of which 18 strains bore mutations in *eis*. Eighteen strains with an MIC of 2.5 μg/ml were predominately sensitive by MGIT (N = 13), of which 4 strains bore the *eis* mutations c(-12)t (N = 3) and g(-10)a.

The a1401g mutation in *rrs* was found in 28 strains that were resistant to both KAN and AMK by both methods. Most of these stains had the maximal MICs used for MycoTB of 40 μg/ml for KAN (N = 22) and 16 μg/ml for AMK. The range of the KAN MICs for the isolates with *eis* mutations partially overlapped with *rrs* a1401g (2.5 to 40 μg/ml), but most of the strains (31 of 40) had MICs of 5 and 10 μg/ml. One isolate with the *rrs* g1484t mutation was resistant to both KAN and AMK by MGIT, but the MIC of the latter was 2 μg/ml, which corresponded to susceptibility (two dilutions from the borderline).

A small proportion of the AMK-resistant strains detected by MGIT (5 of 33) had MICs ranging from 0.5 to 2 μg/ml, which corresponded to susceptibility by the MycoTB method. Four of these strains bore the mutation c(-14)t in *eis*, and one isolate harbored g1484t in the *rrs* gene. Two more isolates with the same c(-14)t mutation were susceptible by MGIT.

### Correlation of the resistant phenotype with the lineage and the patient treatment history

The TB-TEST molecular assay allows the identification of the main genetic lineages based on an analysis of six single nucleotide polymorphisms (SNPs). According to the obtained data, the Beijing genotype prevailed among the investigated strains and was detected in 111/144 (77%) of the cases. Thirty of these strains belonged to the Beijing BO/W148 lineage (Table 3). The proportions of the MDR and XDR MBT among all of the Beijing-strains were 59/111 (53%) and 31/111 (28%), respectively. The MDR plus XDR prevalence among the primary cases was 54% (N = 36 of 67), of which 4 strains possessed the XDR phenotype. In the retreatment and relapse cases, the prevalence of the MDR plus XDR strains was 90% (N = 69 of 77), whereas XDR was detected in 47% (N = 36) of the cases.

**Table 3:** Associations of the drug resistance profile with the strain lineage and the patient history of previous anti-TB drugs.

Isolate profile (MGIT)	Beijing (N = 111, 77%)		Euro-American (N = 33, 23%)				Primary cases (N = 67)		Retreatment cases (N = 58)		Relapse cases (N = 19)	
	BO/ W148	Other Beijing	Haarlem	LAM	Ural	Other EA						
XDR	10	21	0	5	2	2	4	6%	27	47%	9	47%
MDR	18	41	2	2	2	0	32	48%	26	45%	7	37%
DR	2	12	0	0	2	4	12	18%	5	9%	3	16%
S	0	7	1	4	0	7	19	28%	0	0%	0	0%

## Discussion

Recent studies on the mechanism of MTB drug resistance demonstrated that the acquisition of resistance was a time-consuming, adaptive process of selection for successful genetic variants bearing mutations in target loci directly involved in the interaction with the drug and in genes and loci responsible for compensatory mechanisms that diminished the drug’s negative impact on bacterial physiology [1]. Moreover, the level of phenotypic resistance to drugs can vary as a result of the different types of mutations selected and the degree of heterogeneity in the MTB population in the clinical isolates [23]. The proposed use of two [23] and three [10] critical concentrations for the MGIT system provides further improvement for microbiological testing. Less expensive microbiological test systems based on drug serial dilutions allow the identification of the MICs of a wide spectrum of the drugs used for therapy.

In this study, we performed the Sensititre MycoTB alongside the Bactec MGIT 960 and identified common mutations leading to drug resistance using the microarray-based method [20] with clinical isolates from the Moscow region. The selected strains had a strong bias toward drug resistance; nevertheless, a drastic difference in the MDR and XDR prevalence rates was observed in patients with a history of previous treatment. Pan-susceptible isolates were found only in primary cases, and the XDR prevalence in primary cases was as low as 6%; in contrast, the XDR prevalence among the retreatment and relapse cases was 47%.

Good correlations between the two phenotypic methods were observed for most drugs, and the MycoTB MIC distributions of the resistant and susceptible isolates identified using the Bactec MGIT 960 method were distinctive for all of the drugs except EMB, KAN and ETH. However, a noticeable number of isolates with borderline or low-level resistance had discordant susceptibility results for most of the drugs tested.

The possible treatment of tuberculosis caused by isolates with low-level resistance using higher drug doses is a disputable issue in tuberculosis therapy [24, 25]. In addition to the phenotypic and genetic characterization of strains, other in vivo factors, such as the population structure [26, 27], pharmacokinetic/pharmacodynamic parameters [28], permeability of the tuberculosis lesions [29], and adverse reactions, also affect the clinical outcome.

The most frequent substitution found when analyzing resistance to RMP was the S531L substitution, which was associated with high-level resistance [10, 13, 30] and was found in strains resistant to both RMP and RFB with high MICs in most cases. However, several of these isolates had intermediate MICs for RMP and were susceptible to RFB, which was similar to earlier findings [31, 32, 33] and diminished the prognostic value of genetic tests based on *rpoB* locus analysis. In contrast, a portion of the isolates with intermediate resistance and substitutions in codons 516 and 526 were identified as susceptible by Bactec MGIT 960, which was in line with previous reports [12]. Previous reports noted the clinical importance of “disputed” mutations in the *rpoB* gene [34, 35, 36]. Because the standard treatment of these patients might result in a poor clinical outcome [37, 38], the use of higher doses of RMP [25] or substitution with RFB [39] is proposed.

The clinical importance of low-level resistance was also confirmed for the FQs in a murine model. In this model, the sterilizing activity of MFX against the low-level resistant strain with GyrB D500N was nearly the same as the sterilizing activity against the wild-type strain; however, relapse was observed in 30% of the cases [40]. In our set of isolates, the *gyrB* substitutions D500H and N538K led to moderately increased MICs for both OFX and MFX, confirming previous findings [41]. Seven isolates with the *gyrA* A90V substitution were resistant and possessed higher MICs for both FQs than previously reported [42].

Unexpectedly, we obtained a significantly higher correlation between MGIT and MycoTB with EMB (0.79) compared to the previously published value of 0.30 [43]. Generally, the presence of mutations in the *embB* 296–497 codon region increases the resistance of the strain to EMB to varying degrees, which might be lower than the plasma drug level of 5 μg/ml [44]. With respect to our data, we could not conclude that the presence of a particular mutation might lead to the exclusion of EMB from the treatment scheme. For example, 9 of the 29 isolates with the M306V substitution had MICs below the breakpoint, of which 3 isolates were also susceptible by MGIT.

In the case of KAN resistance, a large proportion of the resistant strains identified by MGIT (20 of 73) had MICs of 5 μg/ml, whereas none of the susceptible isolates had the same MICs. If the KAN resistance breakpoint for the MycoTB test system was changed to below 5 μg/ml and resistance was ascribed to these strains, the raw agreement for an MGIT and MycoTB of 0.97 and a Cohen’s kappa of 0.93 were observed instead of 0.83 and 0.65, respectively. Eighteen of these strains bore mutations in the *eis* promoter region, which is associated with a moderate increase in the MIC for KAN [45].

A significant discrepancy was observed for ETH resistance in the microbiological testing. Forty-four percent of the tested isolates had ETH MICs of 2.5 and 5 μg/ml, which were below the breakpoint, although the numbers of strains characterized as resistant and susceptible by MGIT for these MICs were comparable. Analogous discrepant results for the ETH MICs of 2.5, 5, and 10 μg/ml were obtained recently by our group using another set of clinical isolates (N = 89, data not shown).

The cross-correlation of the MICs of two different drugs could indicate common mechanisms of drug resistance or could reflect the history of the ineffective simultaneous use of two drugs. A correlation analysis of the MICs detected by MycoTB revealed an increased correlation in the first-line drug group and a partial increase in the second-line drug group, whereas the average correlation between the groups did not exceed the average correlation among all of the drugs. The only exception to this rule was RFB, where the distribution of the MIC was correlated with RMP and to a lesser degree with STR. If this correlation can be explained by shared mechanisms of resistance formation [31] in the first case, then the correlation might reflect the contribution of some unknown genetic or regulatory factor in the second case. The highest correlation was observed between OFX and MFX and the injectable drugs KAN and AMK, which share drug resistance mechanisms. The methods have some limitations when considering RMP and INH MICs and to a lesser extent RFB due to the truncated range of the MICs used in the MycoTB panel. A large portion of the strains had MICs >16 for RMP, and this group might possibly include strains with MICs of 17 μg/ml and MICs >160 μg/ml.

The identification of the MTB lineage is believed to have a clinical impact due to the association of some lineages, particularly modern Beijing, with virulence [46], transmission [47], drug tolerance [48] and adaptation [49]. The Beijing lineage was identified in 77% of the isolates, including the “successful” B0/W148 sublineage (21%) [50]. Beijing was more associated with the drug-resistant forms than the Euro-American strains. The percentage of Beijing MDR strains was significantly elevated compared to the percentage of MDR strains of the Euro-American lineage (53% vs. 18%); however, the XDR prevalence rates were similar for both clusters (27–28%). No pan-susceptible isolates of the Beijing B0/W148 and Ural lineages were identified.

Although the average correlation between the three analyzed methods was approximately 94% (S3 Table), MycoTB had a slight advantage due to its cost and the amount of information obtained. The effectiveness of the TB-TEST assay application for the analysis of clinical material largely depended on the number of mycobacterial cells in the sample similar to any other molecular method. The TB-TEST works correctly with 96% of AFB-positive and 25% of AFB-negative clinical samples [20]. Thus, in the case of the failure of the direct genetic analysis of a clinical sample, a short-term liquid culture could be used with the Bactec MGIT automated system, and the obtained DNA from the isolate could be re-assayed using the TB-TEST. In this case, the time to obtain the resistance profile will be shortened by the time needed for the second cultivation in the Bactec system, and comprehensive and reliable data concerning the mutation profile and genotype can be obtained.

Nevertheless, the fastest molecular genetic assays demand further improvement to expand the number of drugs analyzed and to reveal the hidden molecular mechanisms of the drug resistance manifestation. The analysis of genomic [51] and post-genomic data via the construction of large-scale databases [52, 53] will likely lead to an improved understanding of MTB physiology and facilitate the development of more informative molecular assays.

## Supporting Information

S1 Table Pairwise correlation coefficients of MIC distributions. (DOC)

S2 Table Correlation of MICs for selected drug pairs. (DOC)

S3 Table Phenotypic characterization of strains with particular substitutions. (DOC)

## References

[1] Trauner A, Borrell S, Reither K, Gagneux S. Evolution of drug resistance in tuberculosis: recent progress and implications for diagnosis and therapy. Drugs. 2014;74(10): 1063–72. 10.1007/s40265-014-0248-y 24962424 PMC4078235

[2] Centers for Disease Control and Prevention (CDC). Emergence of *Mycobacterium tuberculosis* with extensive resistance to second-line drugs--worldwide, 2000–2004. MMWR Morb Mortal Wkly Rep. 2006;55(11): 301–5. 16557213

[3] Haydel SE. Extensively drug-resistant tuberculosis: a sign of the times and an impetus for antimicrobial discovery. Pharmaceuticals (Basel). 2010;3(7): 2268–90. 21170297 10.3390/ph3072268 PMC3002907

[4] World Health Organization. Global Tuberculosis Report 2014. Geneva, Switzerland. Available: http://www.who.int/tb/publications/global_report/gtbr14_main_text.pdf

[5] Jagielski T, Minias A, van Ingen J, Rastogi N, Brzostek A, Zaczek A, et al Methodological and clinical aspects of the molecular epidemiology of *Mycobacterium tuberculosis* and other Mycobacteria. Clin Microbiol Rev. 2016;29(2): 239–90. 10.1128/CMR.00055-15 26912567 PMC4786889

[6] Springer B, Calligaris-Maibach RC, Ritter C, Bottger EC. Tuberculosis drug resistance in an area of low endemicity in 2004 to 2006: semiquantitative drug susceptibility testing and genotyping. J Clin Microbiol. 2008;46(12):4064–7. 10.1128/JCM.01114-08 18923010 PMC2593253

[7] Kim SJ. Drug-susceptibility testing in tuberculosis: methods and reliability of results. Eur Respir J. 2005;25(3): 564–9. 10.1183/09031936.05.00111304 15738303

[8] Lee J, Armstrong DT, Ssengooba W, Park JA, Yu Y, Mumbowa F, et al Sensititre MYCOTB MIC plate for testing *Mycobacterium tuberculosis* susceptibility to first- and second-line drugs. Antimicrob Agents Chemother. 2014;58(1): 11–8. 10.1128/AAC.01209-13 24100497 PMC3910714

[9] Zhang Z, Wang Y, Pang Y, Liu C. Comparison of different drug susceptibility test methods to detect rifampin heteroresistance in *Mycobacterium tuberculosis*. Antimicrob Agents Chemother. 2014;58(9): 5632–5. 10.1128/AAC.02778-14 25022589 PMC4135805

[10] Springer B, Lucke K, Calligaris-Maibach R, Ritter C, Bottger EC. Quantitative drug susceptibility testing of *Mycobacterium tuberculosis* by use of MGIT 960 and EpiCenter instrumentation. J Clin Microbiol. 2009;47(6): 1773–80. 10.1128/JCM.02501-08 19339475 PMC2691107

[11] Hall L, Jude KP, Clark SL, Dionne K, Merson R, Boyer A, et al Evaluation of the Sensititre MycoTB plate for susceptibility testing of the *Mycobacterium tuberculosis* complex against first- and second-line agents. J Clin Microbiol. 2012;50(11): 3732–4. 10.1128/JCM.02048-12 22895034 PMC3486270

[12] Rigouts L, Gumusboga M, de Rijk WB, Nduwamahoro E, Uwizeye C, de Jong B, et al Rifampin resistance missed in automated liquid culture system for *Mycobacterium tuberculosis* isolates with specific rpoB mutations. J Clin Microbiol. 2013;51(8): 2641–5. 10.1128/JCM.02741-12 23761146 PMC3719602

[13] Kambli P, Ajbani K, Sadani M, Nikam C, Shetty A, Udwadia Z, et al Defining multidrug-resistant tuberculosis: correlating GenoType MTBDRplus assay results with minimum inhibitory concentrations. Diagn Microbiol Infect Dis. 2015;82(1): 49–53. 10.1016/j.diagmicrobio.2015.01.009 25749461 PMC4414878

[14] Franzblau SG, Witzig RS, McLaughlin JC, Torres P, Madico G, Hernandez A, et al Rapid, low-technology MIC determination with clinical *Mycobacterium tuberculosis* isolates by using the microplate Alamar Blue assay. J Clin Microbiol. 1998;36(2): 362–6. 9466742 10.1128/jcm.36.2.362-366.1998 PMC104543

[15] van Klingeren B, Dessens-Kroon M, van der Laan T, Kremer K, van Soolingen D. Drug susceptibility testing of *Mycobacterium tuberculosis* complex by use of a high-throughput, reproducible, absolute concentration method. J Clin Microbiol. 2007;45(8): 2662–8. 10.1128/JCM.00244-07 17537932 PMC1951260

[16] Abuali MM, Katariwala R, LaBombardi VJ. A comparison of the Sensititre(R) MYCOTB panel and the agar proportion method for the susceptibility testing of *Mycobacterium tuberculosis*. Eur J Clin Microbiol Infect Di. 2012;31(5): 835–9. 10.1007/s10096-011-1382-z 21866324

[17] Heysell SK, Pholwat S, Mpagama SG, Pazia SJ, Kumburu H, Ndusilo N, et al Sensititre MycoTB plate compared to Bactec MGIT 960 for first- and second-line antituberculosis drug susceptibility testing in Tanzania: a call to operationalize MICs. Antimicrob Agents Chemother. 2015;59(11): 7104–8. 10.1128/AAC.01117-15 26303804 PMC4604373

[18] Zhang Z, Wang Y, Pang Y, Kam KM. Ethambutol resistance as determined by broth dilution method correlates better than sequencing results with embB mutations in multidrug-resistant *Mycobacterium tuberculosis* isolates. J Clin Microbiol. 2014;52(2): 638–41. 10.1128/JCM.02713-13 24478502 PMC3911357

[19] Dominguez J, Boettger EC, Cirillo D, Cobelens F, Eisenach KD, Gagneux S, et al Clinical implications of molecular drug resistance testing for *Mycobacterium tuberculosis*: a TBNET/RESIST-TB consensus statement. Int J Tuberc Lung Dis. 2016;20(1): 24–42. 10.5588/ijtld.15.0221 26688526

[20] Zimenkov DV, Kulagina EV, Antonova OV, Zhuravlev VY, Gryadunov DA. Simultaneous drug resistance detection and genotyping of *Mycobacterium tuberculosis* using a low-density hydrogel microarray. J Antimicrob Chemother. 2016;71(6): 1520–31. 10.1093/jac/dkw015 26929267

[21] World Health Organization. Policy Guidance on Drug-Susceptibility Testing (DST) of Second-Line Antituberculosis Drugs. Geneva: WHO; 2008 Available: http://www.who.int/tb/publications/2008/whohtmtb_2008_392/en/index.html. 26290924

[22] Sharma M, Thibert L, Chedore P, Shandro C, Jamieson F, Tyrrell G, et al Canadian multicenter laboratory study for standardized second-line antimicrobial susceptibility testing of *Mycobacterium tuberculosis*. J Clin Microbiol. 2011 12;49(12): 4112–6 10.1128/JCM.05195-11 21998413 PMC3232997

[23] Bottger EC. The ins and outs of *Mycobacterium tuberculosis* drug susceptibility testing. Clin Microbiol Infect. 2011;17(8): 1128–34. 10.1111/j.1469-0691.2011.03551.x 21631641

[24] Cynamon MH, Zhang Y, Harpster T, Cheng S, DeStefano MS. High-dose isoniazid therapy for isoniazid-resistant murine *Mycobacterium tuberculosis* infection. Antimicrob Agents Chemother. 1999;43(12): 2922–4. 10582883 10.1128/aac.43.12.2922 PMC89588

[25] van Ingen J, Aarnoutse R, de Vries G, Boeree MJ, van Soolingen D. Low-level rifampicin-resistant *Mycobacterium tuberculosis* strains raise a new therapeutic challenge. Int J Tuberc Lung Dis. 2011;15(7): 990–2. 10.5588/ijtld.10.0127 21682979

[26] Streicher EM, Bergval I, Dheda K, Böttger EC, Gey van Pittius NC, Bosman M, et al Mycobacterium tuberculosis population structure determines the outcome of genetics-based second-line drug resistance testing. Antimicrob Agents Chemother. 2012;56(5): 2420–7. 10.1128/AAC.05905-11 22330913 PMC3346650

[27] Sampson SL. Strength in Diversity: Hidden Genetic Depths of *Mycobacterium tuberculosis*. Trends Microbiol. 2016;24(2): 82–4. 10.1016/j.tim.2015.12.006 26755442

[28] Gumbo T. New susceptibility breakpoints for first-line antituberculosis drugs based on antimicrobial pharmacokinetic/pharmacodynamic science and population pharmacokinetic variability. Antimicrob Agents Chemother. 2010;54(4): 1484–91. 10.1128/AAC.01474-09 20086150 PMC2849358

[29] Sarathy JP, Zuccotto F, Hsinpin H, Sandberg L, Via LE, Marriner GA, et al Prediction of Drug Penetration in Tuberculosis Lesions. ACS Infect Dis. 2016;2(8): 552–63 10.1021/acsinfecdis.6b00051 27626295 PMC5028112

[30] Yakrus MA, Driscoll J, Lentz AJ, Sikes D, Hartline D, Metchock B, et al Concordance between molecular and phenotypic testing of *Mycobacterium tuberculosis* complex isolates for resistance to rifampin and isoniazid in the United States. J Clin Microbiol. 2014;52(6):1932–7. 10.1128/JCM.00417-14 24648563 PMC4042757

[31] Bodmer T, Zurcher G, Imboden P, Telenti A. Mutation position and type of substitution in the beta-subunit of the RNA polymerase influence in-vitro activity of rifamycins in rifampicin-resistant *Mycobacterium tuberculosis*. J Antimicrob Chemother. 1995;35(2): 345–8. 7759399 10.1093/jac/35.2.345

[32] Williams DL, Spring L, Collins L, Miller LP, Heifets LB, Gangadharam PR, et al Contribution of rpoB mutations to development of rifamycin cross-resistance in Mycobacterium tuberculosis. Antimicrob Agents Chemother. 1998;42(7): 1853–7. 9661035 10.1128/aac.42.7.1853 PMC105697

[33] Yuen LK, Leslie D, Coloe PJ. Bacteriological and molecular analysis of rifampin-resistant Mycobacterium tuberculosis strains isolated in Australia. J Clin Microbiol. 1999;37(12): 3844–50. 10565894 10.1128/jcm.37.12.3844-3850.1999 PMC85826

[34] Ho J, Jelfs P, Sintchencko V. Phenotypically occult multidrug-resistant *Mycobacterium tuberculosis*: dilemmas in diagnosis and treatment. J Antimicrob Chemother. 2013;68(12): 2915–20. 10.1093/jac/dkt284 23838950

[35] Van Deun A, Aung KJ, Hossain A, de Rijk P, Gumusboga M, Rigouts L, et al Disputed *rpoB* mutations can frequently cause important rifampicin resistance among new tuberculosis patients. Int J Tuberc Lung Dis. 2015;19(2): 185–90. 10.5588/ijtld.14.0651 25574917

[36] Shah NS, Grace Lin SY, Barry PM, Cheng YN, Schecter G, Desmond E. Clinical Impact on Tuberculosis Treatment Outcomes of Discordance Between Molecular and Growth-Based Assays for Rifampin Resistance, California 2003–2013. Open Forum Infect Dis. 2016;3(3): ofw150 10.1093/ofid/ofw150 27704008 PMC5047429

[37] Van Deun A, Aung KJ, Bola V, Lebeke R, Hossain MA, de Rijk WB, et al Rifampin drug resistance tests for tuberculosis: challenging the gold standard. J Clin Microbiol. 2013;51(8): 2633–40. 10.1128/JCM.00553-13 23761144 PMC3719626

[38] Williamson DA, Roberts SA, Bower JE, Vaughan R, Newton S, Lowe O, et al Clinical failures associated with *rpoB* mutations in phenotypically occult multidrug-resistant *Mycobacterium tuberculosis*. Int J Tuberc Lung Dis. 2012;16(2): 216–20. 10.5588/ijtld.11.0178 22137551

[39] Chen HY, Yu MC, Huang WL, Wu MH, Chang YL, Che CR, et al Molecular detection of rifabutin-susceptible *Mycobacterium tuberculosis*. J Clin Microbiol. 2012;50(6): 2085–8. 10.1128/JCM.00652-12 22442316 PMC3372129

[40] Fillion A, Aubry A, Brossier F, Chauffour A, Jarlier V, Veziris N. Impact of fluoroquinolone resistance on bactericidal and sterilizing activity of a moxifloxacin-containing regimen in murine tuberculosis. Antimicrob Agents Chemother. 2013;57(9): 4496–500. 10.1128/AAC.00506-13 23836169 PMC3754299

[41] Malik S, Willby M, Sikes D, Tsodikov OV, Posey JE. New insights into fluoroquinolone resistance in *Mycobacterium tuberculosis*: functional genetic analysis of *gyrA* and *gyrB* mutations. PLoS One. 2012;7(6): e39754 10.1371/journal.pone.0039754 22761889 PMC3386181

[42] Niward K, Angeby K, Chryssanthou E, Paues J, Bruchfeld J, Jureen P, et al Susceptibility testing breakpoints for *Mycobacterium tuberculosis* categorize isolates with resistance mutations in *gyrA* as susceptible to fluoroquinolones: implications for MDR-TB treatment and the definition of XDR-TB. J Antimicrob Chemother. 2016;71(2): 333–8. 10.1093/jac/dkv353 26538509

[43] Banu S, Rahman SM, Khan MS, Ferdous SS, Ahmed S, Gratz J, et al Discordance across several methods for drug susceptibility testing of drug-resistant *Mycobacterium tuberculosis* isolates in a single laboratory. J Clin Microbiol. 2014;52(1): 156–63. 10.1128/JCM.02378-13 24172155 PMC3911413

[44] McIlleron H, Wash P, Burger A, Norman J, Folb PI, Smith P. Determinants of rifampin, isoniazid, pyrazinamide, and ethambutol pharmacokinetics in a cohort of tuberculosis patients. Antimicrob Agents Chemother. 2006;50(4): 1170–7. 10.1128/AAC.50.4.1170-1177.2006 16569826 PMC1426981

[45] Engstrom A, Perskvist N, Werngren J, Hoffner SE, Jureen P. Comparison of clinical isolates and in vitro selected mutants reveals that *tlyA* is not a sensitive genetic marker for capreomycin resistance in *Mycobacterium tuberculosis*. J Antimicrob Chemother. 2011;66(6): 1247–54. 10.1093/jac/dkr109 21427106

[46] Ribeiro SC, Gomes LL, Amaral EP, Andrade MR, Almeida FM, Rezende AL, et al *Mycobacterium tuberculosis* strains of the modern sublineage of the Beijing family are more likely to display increased virulence than strains of the ancient sublineage. J Clin Microbiol. 2014;52(7): 2615–24. 10.1128/JCM.00498-14 24829250 PMC4097719

[47] Gagneux S, Burgos MV, DeRiemer K, Encisco A, Munoz S, Hopewell PC, et al Impact of bacterial genetics on the transmission of isoniazid-resistant *Mycobacterium tuberculosis*. PLoS Pathog. 2006;2(6): e61 10.1371/journal.ppat.0020061 16789833 PMC1479046

[48] de Keijzer J, de Haas PE, de Ru AH, van Veelen PA, van Soolingen D. Disclosure of selective advantages in the "modern" sublineage of the *Mycobacterium tuberculosis* Beijing genotype family by quantitative proteomics. Mol Cell Proteomics. 2014;13(10): 2632–45. 10.1074/mcp.M114.038380 25022876 PMC4188992

[49] McGrath M, Gey van Pittius NC, van Helden PD, Warren RM, Warner DF. Mutation rate and the emergence of drug resistance in *Mycobacterium tuberculosis*. J Antimicrob Chemother. 2014;69(2): 292–302. 10.1093/jac/dkt364 24072169

[50] Mokrousov I, Vyazovaya A, Solovieva N, Sunchalina T, Markelov Y, Chernyaeva E, et al Trends in molecular epidemiology of drug-resistant tuberculosis in Republic of Karelia, Russian Federation. BMC Microbiol. 2015;15: 279 10.1186/s12866-015-0613-3 26679959 PMC4683759

[51] Joshi KR, Dhiman H, Scaria V. tbvar: A comprehensive genome variation resource for *Mycobacterium tuberculosis*. Database (Oxford). 2014;2014: bat083. 24408216 10.1093/database/bat083 PMC3885892

[52] Reddy TB, Riley R, Wymore F, Montgomery P, DeCaprio D, Engels R, et al TB database: an integrated platform for tuberculosis research. Nucleic Acids Res. 2009;37(Database issue): D499–508. 10.1093/nar/gkn652 18835847 PMC2686437

[53] Galagan JE, Sisk P, Stolte C, Weiner B, Koehrsen M, Wymore F, et al TB database 2010: overview and update. Tuberculosis (Edinb). 2010;90(4): 225–35. 20488753 10.1016/j.tube.2010.03.010

