# Supplementary material for: A Comparison of the Sensititre MycoTB Plate, the Bactec MGIT 960, and a Microarray-Based Molecular Assay for the Detection of Drug Resistance in Clinical Mycobacterium tuberculosis Isolates in Moscow, Russia
Source: PLoS One. 2016 Nov 30;11(11):e0167093. doi: 10.1371/journal.pone.0167093 (PMC5130259; doi:10.1371/journal.pone.0167093)
Supplement: S1 Table — (DOC) [file pone.0167093.s001.doc]

## S1 Table. Pairwise correlation coefficients of MIC distributions.

MICs for streptomycin (STR), rifampin (RMP), isoniazid (INH), ethambutol (EMB), rifabutin (RFB), ofloxacin (OFX), moxifloxacin (MFX), kanamycin (KAN), amikacin (AMK), PAS, ethionamide (ETH), and cycloserine (DCS), detected using the Sensititre MycoTB.

|  | STR | RMP | INH | EMB | RFB | OFX | MFX | KAN | AMK | PAS | ETH | DCS |
| --- | --- | --- | --- | --- | --- | --- | --- | --- | --- | --- | --- | --- |
| STR | 1 | 0.56 | 0.43 | 0.35 | 0.36 | 0.15 | 0.2 | 0.22 | 0.25 | 0.22 | -0 | 0.05 |
| RMP | 0.56 | 1 | 0.58 | 0.43 | 0.49 | 0.19 | 0.22 | 0.27 | 0.22 | 0.13 | 0.04 | 0.06 |
| INH | 0.43 | 0.58 | 1 | 0.42 | 0.11 | 0.19 | 0.25 | 0.28 | 0.25 | 0.14 | 0.17 | 0.15 |
| EMB | 0.35 | 0.43 | 0.42 | 1 | 0.16 | 0.15 | 0.2 | 0.31 | 0.27 | 0.12 | 0.12 | 0.13 |
| RFB | 0.36 | 0.49 | 0.11 | 0.16 | 1 | 0.17 | 0.2 | 0.09 | -0 | 0.3 | 0.07 | 0.01 |
| OFX | 0.15 | 0.19 | 0.19 | 0.15 | 0.17 | 1 | 0.86 | 0.31 | 0.22 | 0.4 | 0.25 | 0.02 |
| MFX | 0.2 | 0.22 | 0.25 | 0.2 | 0.2 | 0.86 | 1 | 0.42 | 0.32 | 0.36 | 0.29 | 0.03 |
| KAN | 0.22 | 0.27 | 0.28 | 0.31 | 0.09 | 0.31 | 0.42 | 1 | 0.88 | 0.1 | 0.06 | 0.03 |
| AMK | 0.25 | 0.22 | 0.25 | 0.27 | -0 | 0.22 | 0.32 | 0.88 | 1 | 0.06 | 0 | 0.04 |
| PAS | 0.22 | 0.13 | 0.14 | 0.12 | 0.3 | 0.4 | 0.36 | 0.1 | 0.06 | 1 | 0.29 | 0.09 |
| ETH | -0 | 0.04 | 0.17 | 0.12 | 0.07 | 0.25 | 0.29 | 0.06 | 0 | 0.29 | 1 | 0.4 |
| DCS | 0.05 | 0.06 | 0.15 | 0.13 | 0.01 | 0.02 | 0.03 | 0.03 | 0.04 | 0.09 | 0.4 | 1 |
