# Supplementary material for: A Comparison of the Sensititre MycoTB Plate, the Bactec MGIT 960, and a Microarray-Based Molecular Assay for the Detection of Drug Resistance in Clinical Mycobacterium tuberculosis Isolates in Moscow, Russia
Source: PLoS One. 2016 Nov 30;11(11):e0167093. doi: 10.1371/journal.pone.0167093 (PMC5130259; doi:10.1371/journal.pone.0167093)
Supplement: S2 Table — (DOC) [file pone.0167093.s002.doc]

## S2 Table. Correlation of MICs for selected drug pairs.

Pairs of rifampin (RMP) vs. rifabutin (RFB), ofloxacin (OFX) vs. moxifloxacin (MFX), and isoniazid (INH) vs. ethionamide (ETH) analyzed. Dotted lines separate resistant and susceptible isolates using the 7H10/7H11 critical concentrations.

|  | | RFB | | | | | | | | |
| --- | --- | --- | --- | --- | --- | --- | --- | --- | --- | --- |
|  |  | 0.1 | 0.3 | 0.5 | 1 | 2 | 4 | 8 | 16 | >16 |
| RMP | 0.125 | 11 |  | 1 |  |  |  |  |  |  |
|  | 0.25 | 4 |  |  |  |  |  |  |  |  |
|  | 0.5 | 3 | 1 |  |  |  |  |  |  |  |
|  | 1 |  | 1 |  |  |  | 1 |  |  |  |
|  | 2 | 1 |  |  |  |  | 1 |  |  |  |
|  | 4 |  | 1 |  |  |  |  |  |  |  |
|  | 8 |  |  | 1 |  |  | 1 | 1 |  |  |
|  | 16 |  |  |  |  | 1 |  | 1 |  |  |
|  | >16 | 1 |  | 3 | 7 | 13 | 28 | 34 | 11 | 2 |
| Correlation coefficient r_xy_ = 0.49  ( r_xy_ = 0.70 if truncated RFB MIC is used from 0.1 to >4 ) | | | | | | | | | | |

|  |  | MFX | | | | | | | |
| --- | --- | --- | --- | --- | --- | --- | --- | --- | --- |
|  |  | 0.06 | 0.13 | 0.25 | 0.5 | 1 | 2 | 4 | 8 |
| OFX | 0.25 | 20 | 3 | 2 |  |  |  |  |  |
|  | 0.5 | 8 | 15 | 16 |  |  |  |  |  |
|  | 1 | 1 |  | 18 | 4 |  |  |  |  |
|  | 2 |  |  | 2 | 2 | 1 |  |  |  |
|  | 4 |  |  | 1 | 2 | 10 | 3 | 1 |  |
|  | 8 |  |  |  | 2 |  | 7 | 7 |  |
|  | 16 |  |  |  |  |  | 1 | 6 | 6 |
|  | 32 |  |  |  |  | 1 |  |  | 5 |
| Correlation coefficient r_xy_ = 0.86 | | | | | | | | | |

|  | | ETH | | | | | | | | |
| --- | --- | --- | --- | --- | --- | --- | --- | --- | --- | --- |
|  |  | 0.3 | 0.6 | 1.3 | 2.5 | 5 | 10 | 20 | 40 | 41 |
| INH | 0.031 |  |  | 3 | 1 | 2 |  |  |  |  |
|  | 0.063 |  |  |  | 2 | 2 |  |  |  |  |
|  | 0.125 |  | 1 |  |  |  | 1 |  |  |  |
|  | 0.25 |  |  |  |  |  |  |  | 1 |  |
|  | 0.5 |  |  |  |  |  | 1 |  | 1 |  |
|  | 1 |  |  | 2 |  | 1 |  | 1 |  |  |
|  | 2 |  | 2 |  | 5 | 5 | 5 |  |  |  |
|  | 4 |  |  |  |  | 3 | 1 | 1 |  |  |
|  | 5 | 1 | 2 | 9 | 9 | 27 | 21 | 5 | 7 | 7 |
| Correlation coefficient r_xy_ = 0.17 | | | | | | | | | | |
