# Supplementary material for: A Comparison of the Sensititre MycoTB Plate, the Bactec MGIT 960, and a Microarray-Based Molecular Assay for the Detection of Drug Resistance in Clinical Mycobacterium tuberculosis Isolates in Moscow, Russia
Source: PLoS One. 2016 Nov 30;11(11):e0167093. doi: 10.1371/journal.pone.0167093 (PMC5130259; doi:10.1371/journal.pone.0167093)
Supplement: S3 Table — (DOC) [file pone.0167093.s003.doc]

## Table S3. Phenotypic characterization of strains with particular substitutions.

Sensitivities and specificities of the microarray assay for each drug using MGIT and MycoTB as reference are calculated for each drug. Correlations between the three assays are calculated as the sum of isolates resistant by the three methods and susceptible by the three methods divided by the total number of samples.

| Rifampin |  |  | | | | | | | | | | |  |
| --- | --- | --- | --- | --- | --- | --- | --- | --- | --- | --- | --- | --- | --- |
| *rpoB* substitutions | MGIT 960 | MYCOTB MIC | | | | | | | | | | |  |
|  |  | 0.125 | 0.25 | 0.5 | 1 | 2 | 4 | 8 | 16 | | >16 | |  |
| L511R | R (N=1) |  |  |  |  |  |  | 1 |  | |  | |  |
|  | S (N=0) |  |  |  |  |  |  |  |  | |  | |  |
| D516V | R (N=3) |  |  |  |  | 1 |  |  |  | | 2 | |  |
|  | S (N=0) |  |  |  |  |  |  |  |  | |  | |  |
| D516Y | R (N=0) |  |  |  |  |  |  |  |  | |  | |  |
|  | S (N=2) |  |  | 1 | 1 |  |  |  |  | |  | |  |
| H526R | R (N=3) |  |  |  |  |  |  |  |  | | 3 | |  |
|  | S (N=0) |  |  |  |  |  |  |  |  | |  | |  |
| H526Y | R (N=2) |  |  |  |  |  |  |  |  | | 2 | |  |
|  | S (N=0) |  |  |  |  |  |  |  |  | |  | |  |
| H526D | R (N=1) |  |  |  |  |  |  |  |  | | 1 | |  |
|  | S (N=0) |  |  |  |  |  |  |  |  | |  | |  |
| H526P | R (N=1) |  |  |  |  |  |  |  |  | | 1 | |  |
|  | S (N=0) |  |  |  |  |  |  |  |  | |  | |  |
| H526L | R (N=1) |  |  |  |  |  | 1 |  |  | |  | |  |
|  | S (N=1) |  |  |  | 1 |  |  |  |  | |  | |  |
| H526N | R (N=0) |  |  |  |  |  |  |  |  | |  | |  |
|  | S (N=1) |  |  | 1 |  |  |  |  |  | |  | |  |
| S531L | R (N=93) |  |  |  |  | 1 |  | 2 | 1 | | 89 | |  |
|  | S (N=0) |  |  |  |  |  |  |  |  | |  | |  |
| S531W | R (N=1) |  |  |  |  |  |  |  |  | | 1 | |  |
|  | S (N=0) |  |  |  |  |  |  |  |  | |  | |  |
| L511P D516G | R (N=1) |  |  |  |  |  |  |  | 1 | |  | |  |
|  | S (N=0) |  |  |  |  |  |  |  |  | |  | |  |
| any substitution | R (N=107) |  |  |  |  | 2 | 1 | 3 | 2 | | 99 | |  |
|  | S (N=4) |  |  | 2 | 2 |  |  |  |  | |  | |  |
| wt | R (N=0) |  |  |  |  |  |  |  |  | |  | |  |
|  | S (N=33) | 27 | 4 | 2 |  |  |  |  |  | |  | |  |
| Sensitivity (MGIT) | 100.0% |  |  |  |  |  |  |  |  | |  | |  |
| Specificity (MGIT) | 89.2% |  |  |  |  |  |  |  |  | |  | |  |
| Sensitivity (MYCOTB) | 100.0% |  |  |  |  |  |  |  |  | |  | |  |
| Specificity (MYCOTB) | 89.2% |  |  |  |  |  |  |  |  | |  | |  |
| Correlation between the three assays | 97.2% |  |  |  |  |  |  |  |  | |  | |  |
|  |  |  |  |  |  |  |  |  |  | |  | |  |
| Rifabutin |  |  |  |  |  |  |  |  |  | |  | |  |
| *rpoB* substitution |  | MYCOTB MIC | | | | | | | | | | |  |
|  |  | 0.125 | 0.25 | 0.5 | 1 | 2 | 4 | 8 | 16 | | >16 | |  |
| L511R | (N=1) |  |  | 1 |  |  |  |  |  | |  | |  |
| D516V | (N=3) | 1 |  |  |  | 1 |  |  | 1 | |  | |  |
| D516Y | (N=2) | 1 |  |  |  |  | 1 |  |  | |  | |  |
| H526R | (N=3) |  |  |  |  |  | 1 |  | 2 | |  | |  |
| H526Y | (N=2) |  |  |  |  |  | 2 |  |  | |  | |  |
| H526D | (N=1) |  |  |  |  |  |  |  | 1 | |  | |  |
| H526P | (N=1) |  |  |  |  |  | 1 |  |  | |  | |  |
| H526L | (N=2) |  | 2 |  |  |  |  |  |  | |  | |  |
| H526N | (N=1) | 1 |  |  |  |  |  |  |  | |  | |  |
| S531L | (N=93) | 1 |  | 3 | 7 | 11 | 26 | 36 | 7 | | 2 | |  |
| S531W | (N=1) |  |  |  |  | 1 |  |  |  | |  | |  |
| L511P D516G | (N=1) |  |  |  |  | 1 |  |  |  | |  | |  |
| any substitution | (N=111) | 4 | 2 | 4 | 7 | 14 | 31 | 36 | 11 | | 2 | |  |
| wt | (N=18) | 16 | 1 | 1 |  |  |  |  |  | |  | |  |
| Sensitivity (MYCOTB) | 100.0% |  |  |  |  |  |  |  |  | |  | |  |
| Specificity (MYCOTB) | 64.3% |  |  |  |  |  |  |  |  | |  | |  |
|  |  |  |  |  |  |  |  |  |  | |  | |  |
| Isoniazid |  |  |  |  |  |  |  |  |  | |  | |  |
| *katG inhA* mutations/substitutions | MGIT 960 | MYCOTB MIC | | | | | | | | | | |  |
|  |  | 0.031 | 0.063 | 0.125 | 0.25 | 0.5 | 1 | 2 | 4 | | >4 | |  |
| S315T1 | R (N=75) |  |  | 1 |  |  | 4 | 12 |  | | 58 | |  |
|  | S (N=0) |  |  |  |  |  |  |  | |  | |  | |
| c(-15)t | R (N=8) |  |  |  | 1 | 1 |  | 2 |  | | 4 | |  |
|  | S (N=0) |  |  |  |  |  |  |  |  | |  | |  |
| S315T1 c(-15)t | R (N=28) |  |  |  |  | 1 |  | 2 | 2 | | 23 | |  |
|  | S (N=0) |  |  |  |  |  |  |  |  | |  | |  |
| S315T1 I335V | R (N=5) |  |  |  |  |  |  |  | 3 | | 2 | |  |
|  | S (N=0) |  |  |  |  |  |  |  |  | |  | |  |
| S315T1 W328C | R (N=1) |  |  |  |  |  |  | 1 |  | |  | |  |
|  | S (N=0) |  |  |  |  |  |  |  |  | |  | |  |
| S315T1 I335V c(-15)t | R (N=1) |  |  |  |  |  |  |  |  | | 1 | |  |
|  | S (N=0) |  |  |  |  |  |  |  |  | |  | |  |
| any mutation/substitution | R (N=118) |  |  | 1 | 1 | 2 | 4 | 17 | 5 | | 88 | |  |
|  | S (N=0) |  |  |  |  |  |  |  |  | |  | |  |
| wt | R (N=1) |  |  | 1 |  |  |  |  |  | |  | |  |
|  | S (N=25) | 14 | 10 |  | 1 |  |  |  |  | |  | |  |
| Sensitivity (MGIT) | 99.2% |  |  |  |  |  |  |  |  | |  | |  |
| Specificity (MGIT) | 100.0% |  |  |  |  |  |  |  |  | |  | |  |
| Sensitivity (MYCOTB) | 99.2% |  |  |  |  |  |  |  |  | |  | |  |
| Specificity (MYCOTB) | 96.2% |  |  |  |  |  |  |  |  | |  | |  |
| Correlation between the three assays | 97.9% |  |  |  |  |  |  |  |  | |  | |  |
| Ethambutol |  |  |  |  |  |  |  |  |  | |  | |  |
| *embB* substitutions | MGIT 960 | MYCOTB MIC | | | | | | |  | |  | |  |
|  |  | 0.5 | 1 | 2 | 4 | 8 | 16 | 32 |  | |  | |  |
| N296H | R (N=1) |  |  |  |  | 1 |  |  |  | |  | |  |
|  | S (N=1) |  |  |  | 1 |  |  |  |  | |  | |  |
| M306I1 | R (N=7) |  |  |  | 2 | 5 |  |  |  | |  | |  |
|  | S (N=2) |  |  | 1 | 1 |  |  |  |  | |  | |  |
| M306I2 | R (N=0) |  |  |  |  |  |  |  |  | |  | |  |
|  | S (N=3) |  |  |  | 2 | 1 |  |  |  | |  | |  |
| M306I3 | R (N=0) |  |  |  |  |  |  |  |  | |  | |  |
|  | S (N=1) |  |  |  | 1 |  |  |  |  | |  | |  |
| M306L | R (N=4) |  |  |  |  | 3 |  | 1 |  | |  | |  |
|  | S (N=0) |  |  |  |  |  |  |  |  | |  | |  |
| M306V | R (N=26) |  |  | 1 | 5 | 18 | 1 | 1 |  | |  | |  |
|  | S (N=3) |  |  |  | 3 |  |  |  |  | |  | |  |
| Y319S | R (N=1) |  |  |  |  | 1 |  |  |  | |  | |  |
|  | S (N=0) |  |  |  |  |  |  |  |  | |  | |  |
| Y319C | R (N=1) |  |  |  |  |  | 1 |  |  | |  | |  |
|  | S (N=1) |  |  |  | 1 |  |  |  |  | |  | |  |
| Y319D | R (N=0) |  |  |  |  |  |  |  |  | |  | |  |
|  | S (N=1) |  |  |  | 1 |  |  |  |  | |  | |  |
| D354A | R (N=12) |  |  |  | 1 | 9 | 2 |  |  | |  | |  |
|  | S (N=3) |  |  | 1 | 2 |  |  |  |  | |  | |  |
| G406A | R (N=7) |  |  |  | 1 | 6 |  |  |  | |  | |  |
|  | S (N=2) |  |  |  | 2 |  |  |  |  | |  | |  |
| G406D | R (N=1) |  |  |  |  | 1 |  |  |  | |  | |  |
|  | S (N=0) |  |  |  |  |  |  |  |  | |  | |  |
| G406S | R (N=3) |  |  |  |  |  | 3 |  |  | |  | |  |
|  | S (N=0) |  |  |  |  |  |  |  |  | |  | |  |
| Q497K | R (N=1) |  |  |  | 1 |  |  |  |  | |  | |  |
|  | S (N=0) |  |  |  |  |  |  |  |  | |  | |  |
| Q497P | R (N=0) |  |  |  |  |  |  |  |  | |  | |  |
|  | S (N=1) |  |  | 1 |  |  |  |  |  | |  | |  |
| Q497R | R (N=10) |  |  |  |  | 4 | 6 |  |  | |  | |  |
|  | S (N=3) |  |  |  | 2 | 1 |  |  |  | |  | |  |
| Q497R M306V | R (N=1) |  |  |  |  | 1 |  |  |  | |  | |  |
|  | S (N=0) |  |  |  |  |  |  |  |  | |  | |  |
| any substitution | R (N=75) |  |  | 1 | 10 | 49 | 13 | 2 |  | |  | |  |
|  | S (N=21) |  |  | 3 | 16 | 2 |  |  |  | |  | |  |
| wt | R (N=4) |  |  |  | 1 | 3 |  |  |  | |  | |  |
|  | S (N=44) | 9 | 15 | 13 | 6 | 1 |  |  |  | |  | |  |
| Sensitivity (MGIT) | 94.9% |  |  |  |  |  |  |  |  | |  | |  |
| Specificity (MGIT) | 67.7% |  |  |  |  |  |  |  |  | |  | |  |
| Sensitivity (MYCOTB) | 94.3% |  |  |  |  |  |  |  |  | |  | |  |
| Specificity (MYCOTB) | 59.5% |  |  |  |  |  |  |  |  | |  | |  |
| Correlation between the three assays | 74.3% |  |  |  |  |  |  |  |  | |  | |  |
| Ofloxacin |  |  |  |  |  |  |  |  |  | |  | |  |
| *gyrA gyrB* substitutions | MGIT 960 | MYCOTB MIC | | | | | | | | |  | |  |
|  |  | 0.25 | 0.5 | 1 | 2 | 4 | 8 | 16 | 32 | |  |  |  |
| G88C | R (N=1) |  |  |  |  |  |  |  | 1 | |  | |  |
|  | S (N=0) |  |  |  |  |  |  |  |  | |  | |  |
| A90V | R (N=7) |  |  |  |  | 3 | 4 |  |  | |  | |  |
|  | S (N=0) |  |  |  |  |  |  |  |  | |  | |  |
| S91P | R (N=4) |  |  |  |  | 3 | 1 |  |  | |  | |  |
|  | S (N=0) |  |  |  |  |  |  |  |  | |  | |  |
| D94A | R (N=5) |  |  |  | 1 | 3 | 1 |  |  | |  | |  |
|  | S (N=0) |  |  |  |  |  |  |  |  | |  | |  |
| D94N | R (N=6) |  |  |  |  |  | 1 | 3 | 2 | |  | |  |
|  | S (N=0) |  |  |  |  |  |  |  |  | |  | |  |
| D94G | R (N=19) |  |  |  |  | 2 | 8 | 6 | 3 | |  | |  |
|  | S (N=0) |  |  |  |  |  |  |  |  | |  | |  |
| D94Y | R (N=1) |  |  |  |  |  | 1 |  |  | |  | |  |
|  | S (N=0) |  |  |  |  |  |  |  |  | |  | |  |
| D94V | R (N=2) |  |  |  |  | 2 |  |  |  | |  | |  |
|  | S (N=0) |  |  |  |  |  |  |  |  | |  | |  |
| D500H | R (N=1) |  |  |  |  | 1 |  |  |  | |  | |  |
|  | S (N=0) |  |  |  |  |  |  |  |  | |  | |  |
| N538K | R (N=1) |  |  |  |  | 1 |  |  |  | |  | |  |
|  | S (N=1) |  |  |  | 1 |  |  |  |  | |  | |  |
| A90V D500N | R (N=1) |  |  |  |  |  |  | 1 |  | |  | |  |
|  | S (N=0) |  |  |  |  |  |  |  |  | |  | |  |
| D94A E540D | R (N=1) |  |  |  |  | 1 |  |  |  | |  | |  |
|  | S (N=0) |  |  |  |  |  |  |  |  | |  | |  |
| D94G G509A | R (N=1) |  |  |  |  |  |  | 1 |  | |  | |  |
|  | S (N=0) |  |  |  |  |  |  |  |  | |  | |  |
| D94G T539P | R (N=1) |  |  |  |  |  |  | 1 |  | |  | |  |
|  | S (N=0) |  |  |  |  |  |  |  |  | |  | |  |
| D94N A90V | R (N=1) |  |  |  |  |  |  | 1 |  | |  | |  |
|  | S (N=0) |  |  |  |  |  |  |  |  | |  | |  |
| H70A G88A G509A | R (N=1) |  |  |  |  | 1 |  |  |  | |  | |  |
|  | S (N=0) |  |  |  |  |  |  |  |  | |  | |  |
| S91P D94A | R (N=1) |  |  |  | 1 |  |  |  |  | |  | |  |
|  | S (N=0) |  |  |  |  |  |  |  |  | |  | |  |
| any substitution | R (N=54) |  |  |  | 2 | 17 | 16 | 13 | 6 | |  | |  |
|  | S (N=1) |  |  |  | 1 |  |  |  |  | |  | |  |
| wt | R (N=0) |  |  |  |  |  |  |  |  | |  | |  |
|  | S (N=89) | 25 | 39 | 23 | 2 |  |  |  |  | |  | |  |
| Sensitivity (MGIT) | 100.0% |  |  |  |  |  |  |  |  | |  | |  |
| Specificity (MGIT) | 98.9% |  |  |  |  |  |  |  |  | |  | |  |
| Sensitivity (MYCOTB) | 100.0% |  |  |  |  |  |  |  |  | |  | |  |
| Specificity (MYCOTB) | 96.7% |  |  |  |  |  |  |  |  | |  | |  |
| Correlation between the three assays | 97.9% |  |  |  |  |  |  |  |  | |  | |  |
| Moxifloxacin |  |  |  |  |  |  |  |  |  | |  | |  |
| *gyrA gyrB* substitutions | MGIT 960 | MYCOTB MIC | | | | | | | | |  | |  |
|  |  | 0.063 | 0.125 | 0.25 | 0.5 | 1 | 2 | 4 | 8 | |  |  |  |
| G88C | R (N=1) |  |  |  |  |  |  |  | 1 | |  | |  |
|  | S (N=0) |  |  |  |  |  |  |  |  | |  | |  |
| A90V | R (N=7) |  |  |  |  | 3 | 3 | 1 |  | |  | |  |
|  | S (N=0) |  |  |  |  |  |  |  |  | |  | |  |
| S91P | R (N=4) |  |  |  |  | 1 | 1 | 2 |  | |  | |  |
|  | S (N=0) |  |  |  |  |  |  |  |  | |  | |  |
| D94A | R (N=5) |  |  | 1 | 2 | 1 | 1 |  |  | |  | |  |
|  | S (N=0) |  |  |  |  |  |  |  |  | |  | |  |
| D94N | R (N=6) |  |  |  |  |  | 1 | 2 | 3 | |  | |  |
|  | S (N=0) |  |  |  |  |  |  |  |  | |  | |  |
| D94G | R (N=19) |  |  |  | 1 | 3 | 3 | 7 | 5 | |  | |  |
|  | S (N=0) |  |  |  |  |  |  |  |  | |  | |  |
| D94Y | R (N=1) |  |  |  |  |  |  | 1 |  | |  | |  |
|  | S (N=0) |  |  |  |  |  |  |  |  | |  | |  |
| D94V | R (N=2) |  |  | 1 |  | 1 |  |  |  | |  | |  |
|  | S (N=0) |  |  |  |  |  |  |  |  | |  | |  |
| D500H | R (N=1) |  |  |  | 1 |  |  |  |  | |  | |  |
|  | S (N=0) |  |  |  |  |  |  |  |  | |  | |  |
| N538K | R (N=2) |  |  |  |  | 2 |  |  |  | |  | |  |
|  | S (N=0) |  |  |  |  |  |  |  |  | |  | |  |
| A90V D500N | R (N=1) |  |  |  |  |  |  |  | 1 | |  | |  |
|  | S (N=0) |  |  |  |  |  |  |  |  | |  | |  |
| D94A E540D | R (N=1) |  |  |  |  |  | 1 |  |  | |  | |  |
|  | S (N=0) |  |  |  |  |  |  |  |  | |  | |  |
| D94G G509A | R (N=1) |  |  |  |  |  |  | 1 |  | |  | |  |
|  | S (N=0) |  |  |  |  |  |  |  |  | |  | |  |
| D94G T539P | R (N=1) |  |  |  |  |  | 1 |  |  | |  | |  |
|  | S (N=0) |  |  |  |  |  |  |  |  | |  | |  |
| D94N A90V | R (N=1) |  |  |  |  |  |  |  | 1 | |  | |  |
|  | S (N=0) |  |  |  |  |  |  |  |  | |  | |  |
| H70A G88A G509A | R (N=1) |  |  |  |  | 1 |  |  |  | |  | |  |
|  | S (N=0) |  |  |  |  |  |  |  |  | |  | |  |
| S91P D94A | R (N=1) |  |  |  | 1 |  |  |  |  | |  | |  |
|  | S (N=0) |  |  |  |  |  |  |  |  | |  | |  |
| any substitution | R (N=55) |  |  | 2 | 5 | 12 | 11 | 14 | 11 | |  | |  |
|  | S (N=0) |  |  |  |  |  |  |  |  | |  | |  |
| wt | R (N=0) |  |  |  |  |  |  |  |  | |  | |  |
|  | S (N=89) | 29 | 18 | 37 | 5 |  |  |  |  | |  | |  |
| Sensitivity (MGIT) | 100.0% |  |  |  |  |  |  |  |  | |  | |  |
| Specificity (MGIT) | 100.0% |  |  |  |  |  |  |  |  | |  | |  |
| Sensitivity (MYCOTB) | 100.0% |  |  |  |  |  |  |  |  | |  | |  |
| Specificity (MYCOTB) | 92.7% |  |  |  |  |  |  |  |  | |  | |  |
| Correlation between the three assays | 95.1% |  |  |  |  |  |  |  |  | |  | |  |
| Kanamycin |  |  |  |  |  |  |  |  |  | |  | |  |
| *rrs eis* mutations | MGIT 960 | MYCOTB MIC | | | | | | |  | |  | |  |
|  |  | 0.63 | 1.25 | 2.5 | 5 | 10 | 20 | 40 |  | |  |  |  |
| a1401g | R (N=28) |  |  |  |  | 4 | 2 | 22 |  | |  | |  |
|  | S (N=0) |  |  |  |  |  |  |  |  | |  | |  |
| g1484t | R (N=1) |  |  |  |  | 1 |  |  |  | |  | |  |
|  | S (N=0) |  |  |  |  |  |  |  |  | |  | |  |
| c(-12)t | R (N=8) |  |  | 2 | 5 |  |  | 1 |  | |  | |  |
|  | S (N=1) |  |  | 1 |  |  |  |  |  | |  | |  |
| c(-14)t | R (N=6) |  |  |  | 1 | 1 | 4 |  |  | |  | |  |
|  | S (N=0) |  |  |  |  |  |  |  |  | |  | |  |
| g(-10)a | R (N=13) |  |  | 1 | 9 | 3 |  |  |  | |  | |  |
|  | S (N=0) |  |  |  |  |  |  |  |  | |  | |  |
| g(-37)t | R (N=12) |  |  |  | 3 | 9 |  |  |  | |  | |  |
|  | S (N=0) |  |  |  |  |  |  |  |  | |  | |  |
| any mutation | R (N=68) |  |  | 3 | 18 | 18 | 6 | 23 |  | |  | |  |
|  | S (N=1) |  |  | 1 |  |  |  |  |  | |  | |  |
| wt | R (N=5) |  |  | 2 | 2 | 1 |  |  |  | |  | |  |
|  | S (N=70) | 24 | 34 | 12 |  |  |  |  |  | |  | |  |
| Sensitivity (MGIT) | 93.2% |  |  |  |  |  |  |  |  | |  | |  |
| Specificity (MGIT) | 98.6% |  |  |  |  |  |  |  |  | |  | |  |
| Sensitivity (MYCOTB) | 97.9% | 95.5% |  |  |  |  |  |  |  | |  | |  |
| Specificity (MYCOTB) | 77.1% | 94.7% |  |  |  |  |  |  |  | |  | |  |
| Correlation between the three assays | 81.3% | 93.8% |  |  |  |  |  |  |  | |  | |  |
|  |  |  |  |  |  |  |  |  |  | |  | |  |
| Amikacin |  |  |  |  |  |  |  |  |  | |  | |  |
| *rrs eis* mutations | MGIT 960 CC = 1.0 | MYCOTB MIC | | | | | | | | |  | |  |
|  |  | 0.125 | 0.25 | 0.5 | 1 | 2 | 4 | 8 | 16 | |  |  |  |
| g1484t | R (N=1) |  |  |  |  | 1 |  |  |  | |  | |  |
|  | S (N=0) |  |  |  |  |  |  |  |  | |  | |  |
| c(-12)t | R (N=0) |  |  |  |  |  |  |  |  | |  | |  |
|  | S (N=9) |  | 6 | 1 | 2 |  |  |  |  | |  | |  |
| c(-14)t | R (N=4) |  |  |  | 1 | 3 |  |  |  | |  | |  |
|  | S (N=2) |  | 1 |  |  | 1 |  |  |  | |  | |  |
| g(-10)a | R (N=0) |  |  |  |  |  |  |  |  | |  | |  |
|  | S (N=13) |  | 3 | 7 | 3 |  |  |  |  | |  | |  |
| g(-37)t | R (N=0) |  |  |  |  |  |  |  |  | |  | |  |
|  | S (N=12) |  |  | 6 | 6 |  |  |  |  | |  | |  |
| wt | R (N=2) |  |  | 1 | 1 |  |  |  |  | |  | |  |
|  | S (N=73) | 16 | 34 | 19 | 4 |  |  |  |  | |  | |  |
| a1401g | R (N=28) |  |  |  |  |  |  | 6 | 22 | |  | |  |
|  | S (N=0) |  |  |  |  |  |  |  |  | |  | |  |
| no rrs a1401g  mutation | R (N=7) |  |  | 1 | 2 | 4 |  |  |  | |  | |  |
|  | S (N=109) | 16 | 44 | 33 | 15 | 1 |  |  |  | |  | |  |
| Sensitivity (MGIT) | 80.0% |  |  |  |  |  |  |  |  | |  | |  |
| Specificity (MGIT) | 100.0% |  |  |  |  |  |  |  |  | |  | |  |
| Sensitivity (MYCOTB) | 100.0% |  |  |  |  |  |  |  |  | |  | |  |
| Specificity (MYCOTB) | 100.0% |  |  |  |  |  |  |  |  | |  | |  |
| Correlation between the three assays | 95.1% |  |  |  |  |  |  |  |  | |  | |  |
|  |  |  |  |  |  |  |  |  |  | |  | |  |
